# Exploration of the pathogenesis of nephrotic syndrome and traditional Chinese medicine intervention based on gut microbiota

**DOI:** 10.3389/fimmu.2024.1430356

**Published:** 2024-12-09

**Authors:** Jing Li, Yupei Xu, Tianhao Sun, Xiaotian Zhang, Huimin Liang, Wei Lin, Hangxing Yu, Bo Yang, Hongtao Yang

**Affiliations:** ^1^ Department of Nephrology, First Teaching Hospital of Tianjin University of Traditional Chinese Medicine, National Clinical Research Center for Chinese Medicine Acupuncture and Moxibustion, Tianjin, China; ^2^ Department of Nephrology, Traditional Chinese Hospital of Xiamen, Xiamen, China; ^3^ Department of Nephrology, Chongqing City Hospital of Traditional Chinese Medicine, Chongqing, China

**Keywords:** nephrotic syndrome, gut microbiota, pathogenesis, traditional chinese medicine, gut, gut-kidney axis

## Abstract

Nephrotic syndrome (NS) represents a prevalent syndrome among various chronic kidney disease pathologies and is known for its higher severity and worse prognosis compared with chronic glomerulonephritis. Understanding its pathogenesis and identifying more effective treatment modalities have long been a concern of kidney specialists. With the introduction of the gut–kidney axis concept and the progress in omics technologies, alterations in the gut microbiota have been observed in primary and secondary NS. This link has been extensively researched in conditions such as diabetic nephropathy and immunoglobulin A (IgA) nephropathy. Thus, dysbiosis of the gut microbiota is seen as a crucial contributing factor in NS; however, there is a lack of comprehensive reviews that elucidate the changes in the gut microbiota across different NS conditions and that describe its mechanistic role in the disease. Moreover, serving as an innate regulator of the gut microbiota, traditional Chinese medicine (TCM) has the potential to exert a profound impact on the expression of inflammation-promoting agents, decreasing the levels of endotoxins and uremic toxins. In addition, it strengthens the stability of the intestinal barrier while controlling the metabolic function of the body through its efficient modulation of the gut microbiota. This intricate process yields far-reaching consequences for NS.

## 1 Introduction

Over the past decade, the prevalence of chronic kidney disease (CKD) has exceeded 9.1%, making kidney disease a major public health burden globally (1). Nephrotic syndrome (NS) is a common manifestation across various CKD pathologies that is characterized by the presence of proteinuria, hypoalbuminemia, hyperlipidemia, and edema. Compared with chronic glomerulonephritis, NS presents with a higher severity and a poorer prognosis. NS can be classified into primary or secondary NS. Primary NS includes minimal change disease (MCD), membranous nephropathy (MN), mesangial proliferative glomerulonephritis (MsPGN), membranoproliferative glomerulonephritis, and focal segmental glomerulosclerosis (FSGS), which are diagnosed primarily through kidney biopsy. Secondary NS is often associated with conditions such as diabetic nephropathy, lupus nephritis (LN), and Henoch–Schönlein purpura nephritis (HSPN). NS is prone to relapses and complications such as infections, thrombosis, cardiovascular diseases, and acute kidney injury (2). However, the exact mechanisms underlying NS remain unclear, and current treatment options are limited. Thus, exploring the pathogenesis of NS and identifying relevant treatment approaches for this disease are crucial for alleviating the societal burden of CKD.

The deepening understanding of diseases and the advent of new technologies have prompted us to explore new perspectives on the pathological mechanisms and treatment methods for this condition. Omics technologies have been widely applied in the exploration of biomarkers and the diagnosis, prognosis, pharmacology, and toxicity assessment of various diseases (3–5), offering novel avenues and strategies for patient identification and continued monitoring. In this context, targeted modulation of the gut microbiota has been suggested as a preventive and therapeutic approach for a variety of diseases; however, its potential remains to be further developed. Since the proposition of the gut–kidney axis, research on the gut microbiota has become a major focus, with extensive studies being done in the field of renal disorders. One study has indicated that the gut microbiota in individuals suffering from kidney diseases experiences significant alterations, including NS (6), and plays a vital role in the onset and progression of diseases. To date, the gut microbiota has been confirmed as a diagnostic and therapeutic target for diabetic kidney disease (DKD) and MN, but there is a lack of comprehensive reviews on the gut microbiota in NS.

**Figure 1:**
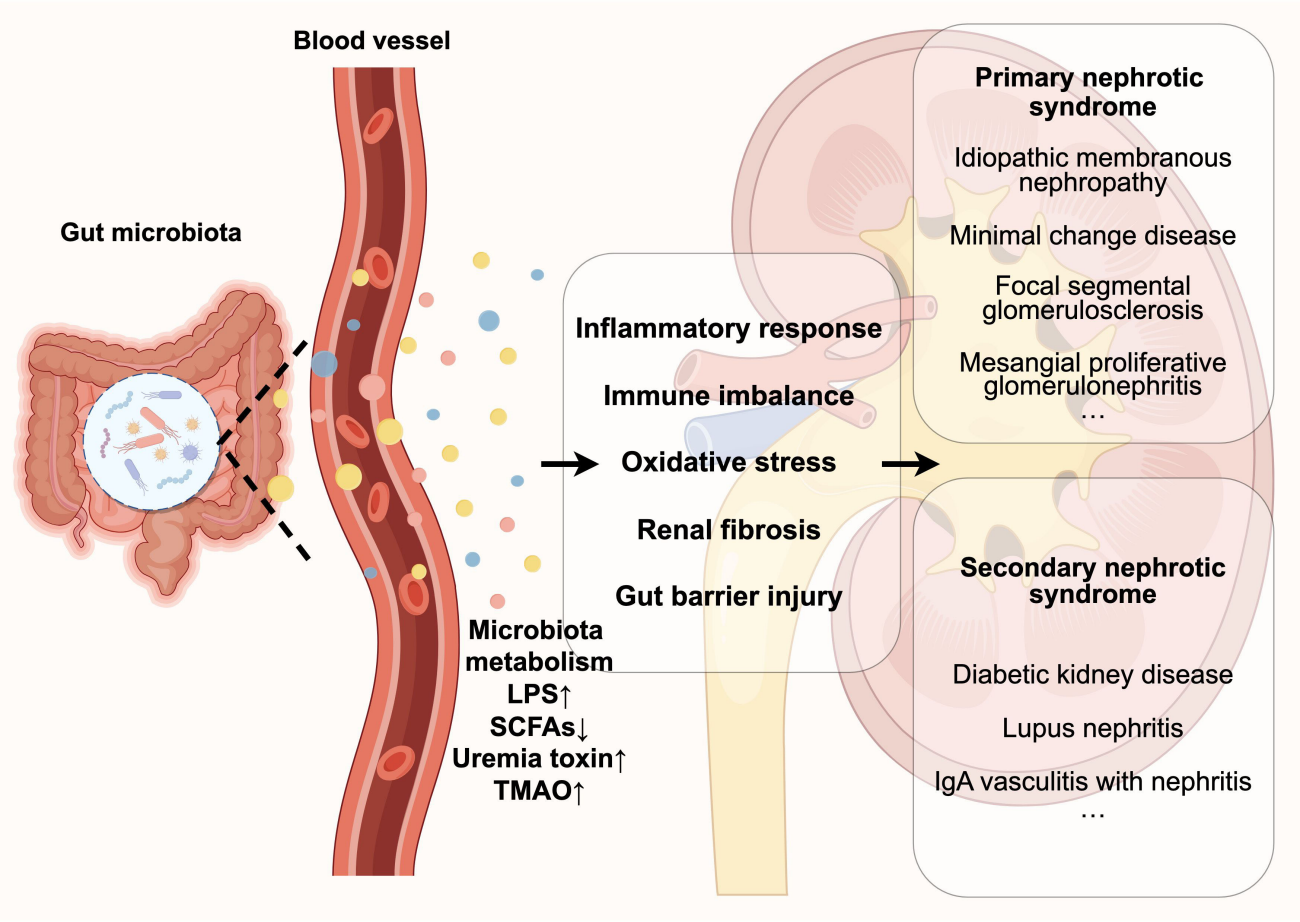
Disturbances in the gut microbiota contribute to the development and progression of nephrotic syndrome by affecting its metabolites (e.g., LPS, SCFAs, etc.), which include triggering a series of pathological reactions such as inflammatory response, immune imbalance, oxidative stress, damage to the gut barrier, and renal fibrosis.

Therefore, this article aimed to review the intrinsic connections between common types of NS and the gut microbiota, attempting to identify potential therapeutic directions for clinical treatment. This study also delves into recent studies concerning how traditional Chinese medicine (TCM) can regulate the gut microbiota as a means of treating NS, aiming to provide more insights into the treatment of NS (**Figure 1**).

## 2 Primary nephrotic syndrome and the gut microbiota

### 2.1 Idiopathic membranous nephropathy

Idiopathic membranous nephropathy (IMN) is a prevalent pathological type of NS in adults that is distinguished by sub-epithelial immune complex deposition, podocyte injury, and diffuse thickening of the glomerular basement membrane. Approximately 30%–50% of patients with IMN will eventually progress to end-stage kidney disease (ESKD) (7). The exact pathogenesis of IMN is yet to be thoroughly deciphered, and to date, it has been mostly considered an antibody-mediated autoimmune disease. The target antigens located on podocytes are recognized by autoantibodies, which then combine with them to form immune complexes and deposit under the basement membrane of podocytes to activate the complement system, thus impairing the function of the glomerular capillary walls and causing podocyte damage and detachment. Consequently, this leads to the increased permeability of the basement membrane and the emergence of large amounts of proteinuria. The immune complexes are composed of various components including immunoglobulin G (IgG) antibodies (mainly IgG4) and the C5b-9 membrane attack complex (8). The identified target antigens include the M-type phospholipase A2 receptor (PLA2R), Nel-like molecule-1, Semaphorin 3B, protocadherin-7, and thrombospondin type 1 domain-containing 7A, all of which bind to IgG subtypes (9).

The gut microbiota can act in a number of ways to impair the body’s immune system. Recent studies on MN and the gut microbiota have shown an increased trend. Studies showed that, compared with the healthy population, patients with IMN have increased levels of *Shigella* spp., *Streptococcus* spp., *Enterobacter* spp., and *Enterococcus* spp. and decreased abundance of *Lachnospira*, Lachnospiraceae, and *Veillonella* (10). *Shigella* spp. are Gram-negative bacilli that produce lipopolysaccharides (LPS) (11), which activate Toll-like receptor 4 (TLR4) and induce the activation of the nuclear factor kappa B (NF-κB) pathway, leading to the production of pro-inflammatory cytokines such as tumor necrosis factor alpha (TNF-α), interleukin 6 (IL-6), and IL-1 (12, 13), which damage podocytes and the glomerular basement membrane. Likewise, a high abundance of *Escherichia coli* increases the circulating levels of certain inflammatory factors (14). It is evident that disturbances in the gut microbiota lead to an imbalance in the expression of these cytokines, which is associated with the pathogenesis of IMN. LPS can stimulate secreted PLA2-IIA (sPLA2-IIA) expression (15). In turn, sPLA2-IIA can exert an action on the immune system by hydrolyzing bacterial membranes and modifying the structure of the gut microbiota, thereby participating in the induction of immune phenotypes and promoting inflammatory responses (16), indicating a link between the PLA2R antigen of IMN and the disturbance of the gut microbiota. In an effort to determine whether the gut microbiota is abnormal in IMN, a number of explorations have been undertaken in succession. Zhang et al. (17) found an increase in *Clostridium* and *Aspergillus*, but a decline in the abundance of the thick-walled phylum in patients with IMN compared with healthy controls. Furthermore, the gut microbiota showed high potential in the differentiation of IMN from DKD, in which patients with IMN had a relatively increased diversity of bacterial α, such as Peptostreptococcaceae_incertae sedis and *Clostridium_sensu_stricto_1*, among others (18), which are also capable of producing LPS and invading into the bloodstream through the compromised intestinal barrier, further reactivating the inflammatory pathway and accelerating the renal inflammatory response (19). In addition, a recent large-scale study (20) included fecal specimens from more than 800 patients. Through clinical studies, animal experiments, and fecal bacterial transplantation techniques, it was identified that those suffering from MN exhibited a reduced abundance of gut microbiota compared with those who were healthy and that it was difficult to construct a model of MN in rats that had been cleared of gut microbiota, suggesting that the naturally occurring microflora in the intestinal tract may be imperative for the pathogenesis of MN. On this basis, Ma et al. (21) and Feng et al. (22) identified several bacterial groups with a causative relationship with the occurrence of MN, among which *Oscillibacter* showed a protective causal relationship in reducing MN occurrence in both studies. Studies have shown that an imbalance between the T helper cell 17 (Th17) and regulatory T cell (Treg) ratio is tightly linked to the pathogenesis of IMN (23). Short-chain fatty acids (SCFAs), which consist of propionate, butyrate, and acetate, are metabolic products of the gut microbiota. SCFAs can exert anti-inflammatory effects by promoting cytokine IL-10 synthesis through binding to specific G-protein-coupled receptors (GPRs), such as GPR43 and GPR109a, in order to induce the production and differentiation of Tregs (24, 25). The study noted that patients with IMN had significantly lower contents of propionic and butyric acids in their feces than the healthy controls (17). Butyrate could enhance the acetylation of the forkhead box protein 3 (Foxp3) promoter and histone H3 in the conserved non-coding region or bind to a free fatty acid receptor to stimulate the proliferation and differentiation of initial CD4^+^ T cells to Tregs (26, 27). Butyrate and propionate can inhibit the expression of cytokines induced by LPS, such as IL-6 and IL-12/p40, thereby protecting the integrity of podocytes and the glomerular basement membrane (28). It has been found that Clostridia can induce intestinal epithelial cells to produce transforming growth factor beta (TGF-β), providing a favorable environment for the formation of Tregs (29).

In summary, the onset and the progression of IMN are highly connected to the gut microbiota and its metabolic metabolites. However, a point of concern is that, due to the complexity of the gut microbiota, the possible etiology of MN cannot be definitively attributed solely to the dysregulation of a singular bacterium (30). Hence, it is imperative to give due attention to the systematic study of the gut microbiota in order to steer clear of any partiality and attain holistic control.

### 2.2 Minimal change disease

In children and adolescents, MCD is the most frequent cause of NS type. It is characterized by lipid accumulation in the renal tubular epithelial cells and the urine, with extensive podocyte foot process effacement observable under electron microscopy. The underlying pathogenesis of MCD is complex and not fully elucidated yet. Most patients can achieve remission with aggressive treatment, but relapses and steroid dependency are common. Deficiency of Tregs assumes a fundamental position in the emergence and recurrence of MCD (31, 32). Recently, a “second hit” hypothesis has been proposed for MCD, suggesting that an imbalance between the ratio of Tregs and CD80 molecules is closely associated with MCD (33). Moreover, several studies have indicated that the slit-diaphragm molecular component nephrin is associated with the occurrence of MCD (34, 35). Animal experiments have shown that overexpressed CD80 and Neph1 interact through their extracellular domains, disrupting the slit diaphragm (36). LPS induces the loss of foot processes by activating TLR-4 to induce CD80 expression, which ultimately leads to increased glomerular filtration barrier permeability and proteinuria (37, 38).

Jiang et al. (39) analyzed the fecal samples from 45 individuals suffering from MCD. Their findings revealed a noteworthy contrast when compared with those of healthy individuals. Specifically, patients with MCD exhibited a notable increase in the abundance of *Bacteroides*, while the levels of *Faecalibacterium* and *Prevotella* were notably decreased. Analogously, Zhang et al. (40) revealed that untreated MCD patients had a reduced gut microbiota alpha diversity, with decreased abundance of *Bacteroides*, *Faecalibacterium*, and *Prevotella*, with the latter showing the most significant changes and pathogenic bacteria such as *Escherichia*–*Shigella* increasing in abundance. After treatment, the composition of the gut microbiota could be partially restored, indicating that the disordered composition of the gut microbiota might be related to the pathogenesis and severity of MCD. *Faecalibacterium* is one of the bacteria that produce butyrate, and it is possible that the reduction of butyrate could facilitate the entry of inflammatory factors into the bloodstream (41). In conclusion, there is an essential connection between MCD and the gut microbiota; however, the mechanisms linking changes in the gut microbiota to kidney damage are not clear and require further investigation.

### 2.3 Focal segmental glomerulosclerosis

FSGS is a pathological change characterized by segmental sclerosis of some glomeruli observed under light microscopy, involving progressive structural changes such as podocyte apoptosis, glomerular basement membrane damage, cellular structure changes, and mesangial matrix proliferation. The central aspect of the pathology of FSGS is podocyte injury (42). Primary FSGS can cause rapid and extensive damage to podocytes, with a sudden clinical onset and extensive podocyte lesions observed under electron microscopy, where over 80% of the capillary surface shows foot process effacement (43).

Its pathogenesis is still being explored, and it has been found that the gut microbiota may have a certain role in it. Zha et al. (44) found that the administration of adriamycin (ADR) inhibited the expression of GPR41 in the renal tissue of an ADR-induced FSGS rat model, whereas an increase in SCFAs upregulated the GPR41 receptor expression, reduced the release of TNF-α and IL-1β, and inhibited the excessive proliferation and abnormal apoptosis of glomerular cells. Shi et al. (45) constructed a rat FSGS model through unilateral nephrectomy and caudal vein injection of ADR. Fecal analysis revealed a distinct increase in the populations of *Bifidobacterium*, *Collinsella*, and *Candida* and a notable decrease in the abundance of *Granulicatella* and *Christensenella*. Maier et al. (46) induced an adult mouse model of FSGS with podocyte-specific gene *Epb41l5* knockout using doxycycline and identified an impaired gut microbiota diversity, i.e., significant increases in Bacteroidetes and *Alistipes* and decreases in *Pseudoflovonifractor* and *Anaerotignum*. Bacteroidetes and *Alistipes* can produce harmful metabolic products such as ammonia, hydrogen sulfide, cresol, and indole (47, 48). The formation of ammonia increased the intestinal pH value, further promoting gut microbiota dysbiosis and disrupting the tight junctions of the intestinal epithelium, leading to endotoxemia (49). It has been demonstrated that indoxyl sulfate (IS) could activate the mammalian target of rapamycin complex 1 (mTORC1), inducing renal fibrosis and promoting CKD progression through a microbiota-dependent mechanism (50, 51). Injection of ADR induced mice to exhibit mesangial proliferation and segmental glomerulosclerosis, a remarkable increase in the urinary albumin/creatinine ratio, and an obvious modification of the gut microbiota, which was mainly reflected in the enrichment of Odoribacter and the reduction of *Turicibacter*, *Marvinbryantia*, and *Rikenella* (52). Furthermore, it dramatically enhanced the serum inflammatory factors, such as the IL-2 level, in BALB/C mice, which could be attributed to the disorganization of the gut microbiota (52). Zhi et al. (53) treated a patient with FSGS using fecal microbiota transplantation (FMT). After treatment, the kidney function of the patient remained stable and the urinary protein decreased, with no relapse after reducing glucocorticoids. It is evident that the pathogenesis of FSGS is closely associated with the gut microbiota. However, current research on FSGS and the gut microbiota has primarily focused on animal experiments, with limited studies on humans.

### 2.4 Mesangial proliferative glomerulonephritis

The characteristic pathology of MsPGN manifests as proliferation of glomerular mesangial cells and deposition of the glomerular extracellular matrix (54). The pathogenesis of MsPGN has not been fully elucidated, but is thought to be related to the impaired clearance of immune complexes, dysfunction in immune regulation, and excessive proliferation of mesangial cells (55).

He et al. (6) observed changes in the microbial communities in cases of MsPGN and MN. Compared with the MN group, increases were noted in the populations of *Bradyrhizobium*, *Hyphomicrobium*, Bradyrhizobiaceae, Hyphomicrobiaceae, and other taxa. Decreases were observed in Ruminococcaceae, *Alistipes*, *Lachnospira*, Tyzzerella, and chloroplast, among others. This research pointed to the underlying change in the gut microbiome that is relevant to MsPGN. Unfortunately, there are only a handful of studies exploring the gut microbiota in MsPGN, which leaves its exact involvement unclear.

## 3 Secondary nephrotic syndrome and the gut microbiota

### 3.1 Diabetic kidney disease

Among the complications associated with diabetes mellitus (DM), DKD stands out as a particularly common microvascular complication that accounts for 20.7% of cases (56, 57). This condition can involve various lesions, such as the glomeruli, tubules, and the interstitium, among others (58). In addition, DKD is a leading contributor to end-stage renal disease (ESRD), which comprises approximately 47% of the ESRD cases in the US (59). Clinical signs of DKD include edema, persistent proteinuria, hypertension, and a progressive reduction in the glomerular filtration rate. The development of DKD involves intricate mechanisms, posing a significant risk of progression to ESRD.

Currently, there is a lack of highly efficacious therapies to prevent renal damage, which underscores the necessity of delving into novel treatment modalities and targets. Emerging research increasingly indicates that the gut–kidney axis is pivotal in the progression of DKD (60). Gut microbiota disturbances can occur as a result of a high-sugar and high-fat environment, dietary habits, medication, and gastrointestinal edema. There is a considerable dysbiosis of the gut microbiota in patients with DKD, which is marked by a decline in beneficial bacteria and an increase in detrimental and pathogenic bacteria. The comparison of patients with DKD and healthy individuals revealed a discernible decrease in the diversity and abundance of gut microbiota, with greater levels of Actinobacteria at the phylum level and, at the class level, a more prevalent abundance of Actinobacteria, Bacilli, and Coriobacteriia and lower abundance of Alphaproteobacteria and Clostridia (61). In contrast to DM patients without renal impairment, those with DKD exhibited notably elevated levels of *Christensenella*, *Clostridium-XIVa*, and *Eisenbergiella*, with *Eisenbergiella* demonstrating a positive correlation with both glomerulosclerosis and thickening of the glomerular basement membrane (62).

Furthermore, it has been found that different stages of DKD exhibit different gut microbial profiles, with *Agathobacter* potentially serving as a biomarker for distinguishing the early and late stages of DKD (63). A meta-analysis indicated that, compared with healthy controls, patients with DKD had fewer butyrate-producing genera such as *Butyricicoccus*, *Faecalibacterium*, *Lachnospira*, and *Roseburia* and that, compared with DM, DKD had a significant enrichment of harmful bacteria such as *Hungatella*, *Bilophila*, and *Escherichia* (64). Among these, *Hungatella* could act on DKD by affecting trimethylamine-*N*-oxide (TMAO). *Clostridium hathewayi* was found to produce trimethylamine, the precursor of TMAO (65). Furthermore, *C. hathewayi* originates from the genus *Hungatella*. Elevated concentrations of TMAO have been demonstrated to instigate renal impairment by stimulating inflammation, oxidative stress, and fibrosis (66). Moreover, a number of immune-related conditions (67), including rheumatoid arthritis (68), type 2 diabetes (69), inflammatory bowel disease, and colorectal cancer (70), have been linked to *Bilophila*, a sulfate-reducing bacteria with pro-inflammatory qualities. *Escherichia*, a potential pathogen, metabolizes dietary tryptophan into precursors of the uremic toxin indole, which further transforms into IS, participating in the process of renal function damage (71). A research indicated a decline in the population of microbiota responsible for the production of butyrate in individuals with DKD, leading to the decreased levels of serum butyrate and total SCFAs (72). SCFAs possess the ability to prevent the activation of the NF-κB pathway, which in turn diminishes the expression of pro-inflammatory cytokines such as TNF-α and IL-6 (73). In addition, under hyperglycemic conditions, SCFAs can exhibit the ability to modulate inflammation in renal tubular cells and podocytes and engage with GPR43 or GPR109A to regulate immunity, consequently lowering the expression of fibrotic and inflammatory genes associated with DKD. This cascade of effects culminates in the mitigation of podocyte injury, as well as mesangial fibrosis, thereby hindering the progression of DKD (74, 75). Studies have demonstrated that the administration of butyrate effectively prevented kidney damage in diabetic rats by enhancing glomerular hypertrophy and mitigating the production of collagen IV and fibronectin (72).

In addition, oxidative stress has emerged as a significant risk factor contributing to the progression of DKD. Sodium butyrate has been revealed to stimulate nuclear factor erythroid 2-related factor 2 (Nrf2) by inhibiting histone deacetylase (HDAC) activity, thus suppressing the renal oxidative stress response and delaying kidney fibrosis (76). Imbalance in the gut microbiota and exposure to a high-glucose environment could weaken the integrity of the intestinal epithelial barrier, allowing bacteria and enterogenic endotoxins to migrate and accelerate kidney damage in diabetic mice (77). By modulating the levels of synaptopodin and claudin-1, butyrate could maintain the integrity of the intestinal barrier (78).

Studies have increasingly found that new diabetes drugs, such as englitazone (79) and saxagliptin/valsartan (80), can exert their effects partly through modulating the gut microbiota. The gut microbiota is essential for the regulation of glucose and lipid metabolism, alleviating inflammatory and host immunity, responding to inflammatory and oxidative stress, and repairing the intestinal barrier, thereby potentially ameliorating kidney function impairment and offering a fresh avenue for the treatment of DKD.

### 3.2 Lupus nephritis

LN is the most prevalent complication of systemic lupus erythematosus (SLE) and is one of the main causes of death from this condition, with roughly 20% of patients inevitably progressing to ESRD (81). Complement activation, inflammation, and immune complex deposition are involved in the pathophysiology of both acute and chronic renal damage in LN. Research on changes in the gut microbiota and their relationship with autoimmune illnesses has exploded in the last few years, including LN. Compared with healthy adults, patients with LN typically exhibit a reduced alpha diversity in their gut microbiota and a lower ratio of Firmicutes/Bacteroidetes; nonetheless, the salient characteristics may include enrichment of the phylum Proteobacteria, genera *Streptococcus* and *Lactobacillus*, and species *Ruminococcus gnavus* and *Lactobacillus reuteri* (82).

Specific LN microbial communities may participate in disease progression by affecting the total quantity of T cells in peripheral blood, the inflammatory responses, and the intestinal barrier stability. According to a cohort study, the serum anti-RG strain-restricted antibodies and the lupus disease activity positively correlated with the relative abundance of *R. gnavus* (83). The study by Zhang et al. (84) established a correlation between the percentage of *R. gnavus* with the absolute numbers of CD4^+^ T-cell subgroups and lymphocyte subgroups in peripheral blood. Notably, significant correlations emerged between the ratios of Th1/Th2 and Th17/Tregs. Another study (85) demonstrated increased intestinal permeability in LN mice. After treatment with five *Lactobacillus* strains, it was found that the damaged intestinal barrier in mice was repaired, with the anti-inflammatory and nephroprotective effects exerted by inhibiting the expression of IL-6, increasing the concentration of IL-10, inhibiting the kidneys from accumulating IgG2a, and by increasing the Treg/Th17 ratio.

The investigation by Valiente et al. (86) revealed that the colonization of NZM2410 mice by segmented filamentous bacteria resulted in a heightened intestinal permeability, along with the amplification of Th17 and lymphoid cells within the lamina propria of the small intestine, and resulted in a more severe case of glomerulonephritis than in mice that were not colonized. The onset of SLE is mostly driven by immunological tolerance rupture and Th17/Treg equilibrium alteration (87). Tregs can not only foster immune tolerance through suppressing autoreactive lymphocytes but can also regulate the production of antinuclear antibodies and the advancement of nephritis in animals with lupus by blocking the development of aberrant B cells (88). A study found that the percentage of Tregs in patients with LN is much lower than that in healthy controls and in non-LN SLE patients (89), and increases in the level of Tregs might be an independent protective factor for the long-term renal prognosis in patients with LN (90).

The inflammatory response is also a major aspect of the gut flora that influences LN. The elevation of the serum inflammatory cytokine IL-6 level correlates with SLE activity (91). By inducing B-cell maturation and differentiation into plasma cells, IL-6 stimulates the generation of autoantibodies. Moreover, during IL-6 stimulation, CD4 T cells might develop into pro-inflammatory Th17 subgroups, which in turn suppress the function of Tregs (92). By downregulating the expression of the pro-inflammatory cytokine IL-6 and upregulating the expression of the anti-inflammatory cytokine IL-10, the gut microbiota can mitigate renal inflammatory responses. It also increases the quantity of Tregs, which can inhibit the formation of aberrant immune complexes and limit renal damage.

### 3.3 Immunoglobulin A vasculitis with nephritis

Immunoglobulin A vasculitis (IgAV), commonly known as Henoch–Schönlein purpura (HSP), is a type of small vessel vasculitis that mainly affects children and is identified by IgA1 immune deposits. It clinically manifests with involvement of the kidneys, gastrointestinal tract, skin, and joints, with a small proportion affecting the lungs and central nervous system (93). When the kidneys are involved, it leads to Henoch–Schönlein purpura nephritis (HSPN), or IgAV-N, the most severe long-term complication of HSP and a major cause of death (94). Approximately 40% of children with IgAV will go on to develop nephritis (95), and adult patients tend to have more severe renal involvement and poorer prognosis compared with children. The clinical manifestations include hematuria, proteinuria, and nephritic syndrome, and patients with nephritic syndrome present with more severe renal lesions and a higher risk of progressing to ESRD (96).

Studies have suggested that the pathogenesis and characteristics of IgAV-N are extremely similar to those of IgA nephropathy (IgAN), with circulating galactose-deficient IgA1 (Gd-IgA1) immune complexes being a common major pathogenic factor for both conditions (97). IgAN is considered to be related to abnormal mucosal immune responses (98). Comparison with healthy controls has demonstrated that patients with IgAV-N and IgAN have remarkably higher levels of circulating zonulin and Gd-IgA1 (99). Zonulin can regulate the intestinal barrier permeability, and increased levels of zonulin can further enhance circulating autoantibodies and inflammation in the gastrointestinal tract and systemically (100). A study indicated that the pathophysiology of IgAV-N may potentially include gut mucosal immunity (99). IgAV is closely related to respiratory and gastrointestinal infections (101), and individuals with gastrointestinal involvement have a higher risk of developing nephritis (102, 103). The current literature demonstrated that children with IgAV have reduced gut microbiota biodiversity (104, 105) and that enrichment of *Aspergillus* and *Actinobacteria* is associated with organ involvement in IgAV (106).

In addition, according to research (107), individuals with IgAV-N had dysbiosis of the gut microbiota, which was primarily marked by an excess of potential pathogens and a decrease in beneficial bacteria. At the genus level, there was a higher relative abundance of *Lactobacillus*, *Shigella*, and *Streptococcus*, but a lower abundance of *Prevotella_9*, with *Streptococcus* showing a positive correlation with hypoalbuminemia and hematuria. Studies have shown that an increase in potential pathogens and a decrease in beneficial bacteria are the main features of the dysbiosis of the gut microbiota in patients with IgAV-N. At the genus level, the abundance of *Bacteroides* was found to be higher, while that of *Prevotella* was lower. *Streptococcus* can enter the bloodstream, trigger macrophage activation, activate mucosal immunity, induce systemic and renal inflammation, and accelerate kidney damage (108). Research found that *Streptococcus* could exacerbate nephritis damage by modulating the chemotaxis of Th22 cells and by stimulating the production of inflammatory cytokines (109). *Escherichia*–*Shigella* can increase the levels of LPS in the bloodstream (110), which activates peripheral B lymphocytes, triggering systemic immunity and inflammation (111); moreover, the exotoxin it produces can directly destroy glomerular endothelial cells and tubular epithelial cells (112). In the late stage of CKD, *Escherichia*–*Shigella* are highly expressed, possibly participating in renal function damage through the excessive production of IS (113). *Prevotella_9* is considered a beneficial bacterium, and its reduction could lead to the decreased production of butyrate (114), which inhibits the generation of pro-inflammatory cytokines and maintenance of the intestinal barrier function (115–117). The gut microbiome and mucosal immunity are closely related. Through both T-cell-dependent and T-cell-independent pathways, dysbiosis of the gut microbiome can also impair the intestinal barrier, cause aberrant immunological activation, increase the antigen burden, and impact the maturation and differentiation of B cells in gut-associated lymphoid tissues. This induces plasma cells to produce large amounts of Gd-IgA1, which forms immune complexes with the autologous antibodies IgG/IgA, depositing under the endothelium, in small vessel walls, and in mesangial areas (98). In this context, they could stimulate the growth of mesangial cells and the extracellular matrix, leading to the migration of neutrophils and T lymphocytes and initiating the complement cascade, which would ultimately result in the release of inflammatory mediators that cause glomerular damage (118, 119).

## 4 Present situation and advantages of traditional Chinese medicine in the treatment of NS

NS is a clinical syndrome caused by glomerular diseases with multiple etiologies and pathological changes and is often accompanied by complications such as thrombosis and infection, which can eventually progress to ESRD. Clinical treatment mainly includes diuretics, hormones, and immunosuppressants, but the long-term use of hormones and immunosuppressants will increase the risk of infection. Some patients have steroid dependence, frequent recurrence after remission, and steroid resistance (2, 120, 121). Therefore, the rational choice of drug regimen aids in prolonging the remission time and improving the prognosis to a certain extent.

The typical symptom of NS is edema. According to the theory of Chinese medicine, NS is an edema disease. As early as thousands of years ago, “Huangdi Neijing” realized that its induction and progressive factors were due to the imbalance of the lung, spleen, and kidney, leading to abnormal water metabolism. Later generations divided the nature of edema into yin shui and yang shui.

In recent years, there have been more and more studies on the treatment of NS with TCM, and the efficacy of TCM has gradually been recognized. The combination of TCM with conventional treatment can enhance the therapeutic effect. The results of a systematic review that included seven articles involving 487 participants with NS confirmed that Huai Qi Huang granules combined with hormone therapy could reduce the recurrence rate, the incidence of infection in children with NS, and the dose of prednisone, Huai Qi Huang granules could reduce the recurrence rate and the incidence of infection, and improve immune function (122). The “Shulifenxiao” formula can be used as an alternative treatment option for steroid- and immunosuppressant-resistant NS of refractory IMN, which has good safety (123). During the Han Dynasty, Zhongjing Zhang recorded several prescriptions for the treatment of kidney diseases in the book “*Shanghan Lun*,” such as the Linggui Zhugan (124), Fangji Huangqi (125, 126), and Zhenwu decoctions (127), which are still in use today. The Mahuang Fuzi and Shenzhuo decoction (MFSD) is a compound prescription summarized by Professor Liu Baoli according to the idea of Zhang Zhongjing in the book “*Jingui Yaolue*.” It consists of six TCMs: Ma Huang (*Ephedra sinica* Stapf.), Hei Fu Zi (*Aconitum carmichaeli* Debx.), Zhi Gan Cao (*Glycyrrhiza uralensis* Fisch.), Gan Jiang (*Zingiber officinale* Rosc.), Fu Ling [*Poria cocos* (Schw.) Wolf], and Chao Bai Zhu (*Atractylodes macrocephala* Koidz.). A multicenter prospective trial included 198 patients with IMN administered MFSD. The study confirmed the effectiveness of MFSD in treating MN. After 24 months, the remission rate was 58.6% (116/198) and the remission rate was 70% (91/130) in the 130 participant subgroups followed up for 36 months (128).

## 5 TCM treats NS by intervening with the gut microbiota

An unbalanced gut microbiota has been reported in patients with NS, with the gut–kidney axis being important in the development and recurrence of the disease (129, 130). On one hand, this dysbiosis of the gut microbiota, typified by a disequilibrium between pathogenic and probiotic bacteria and a compromised intestinal epithelial barrier, facilitates the bloodstream entrance of uremic toxins, LPS, bacterial endotoxins, and pathogenic bacteria. Furthermore, the gut microbiota interferes with the production of metabolic substances and activates inflammatory signaling pathways and cytokines, thereby triggering immune inflammation and fibrosis processes in nephritis. Probiotic intervention has the potential to counter this imbalance in the gut microbiota (131). Increasing evidence has demonstrated that TCM exhibits unique therapeutic effects in patients with NS (132), acting through multiple levels, targets, and pathways on the pathologic process of NS (133–135). The gut microbiota, as one of the targets of TCM, influences the disease progression through the systemic action of the gut–kidney axis. Current clinical research indicates that TCM affects NS through the following key links by acting on the gut microbiota.

### 5.1 Suppressing the expression of pro-inflammatory cytokines

Inflammation is a crucial contributing factor to kidney damage and fibrosis in renal diseases (136), and suppression of the inflammatory responses is key to stopping the progression of NS. The control of the host’s inflammatory immune system is greatly impacted by the reorganization of the gut microbiome. By increasing the abundance of *Akkermansia* while simultaneously reducing the abundance of *Klebsiella*, Sanziguben polysaccharides (SZPs) have demonstrated efficacy in curing disorders in the gut microbiota found in DKD mice. This further alleviates renal inflammation by blocking the TLR4/NF-κB/NLRP3 pathway, effectively lowering the expression levels of TLR4, phosphorylated NF-κB p65, NLRP3, and the interleukins IL-18 and IL-1β (137). Fufang Zhenzhu Tiaozhi (FTZ) capsule, which is composed of Chinese medicinal herbs [i.e., *Coptis chinensis* Franch., *Ligustrum Lucidum* W.T. Aiton, *Cirsium japonicum* DC., *Salvia miltiorrhiza* Bunge, *Panax notoginseng* (Burkill) F.H. Chen, *Eucommia ulmoides* Oliv, *Citrus medica* L., and *Atractylodes macrocephala* Koidz.], is a patented Chinese medicine compound preparation developed by Professor Guo Jiao according to the theory of “Tiaogan Qishu Huazhuo.” It has anti-inflammatory effects, improving the glucose and lipid metabolism, intestinal metabolites, and the gut microbiota (138). It is mainly used in the clinical treatment of DKD (139) and non-alcoholic fatty liver (140). FTZ polysaccharides (FTZPs) comprise the main active ingredient. Lan et al. (141) found that FTZPs could ameliorate the gut microbiota in DKD mice and diminish the aberrant proliferation of *Weissella*, *Enterococcus*, and *Akkermansia*, which exhibit a positive link to markers of kidney impairment. In DKD mice, continuous administration of FTZPs could also dramatically lower the expression of the pro-inflammatory cytokines chemokine (C–C motif) ligand 2, IL-6, and intercellular adhesion molecule-1 (ICAM-1). Moreover, transcriptomic analysis showed a reduction in the expression of the kidney genes linked to inflammation. Resveratrol has the characteristics of anti-oxidative stress, anti-inflammation, and anti-apoptosis (142). Similarly, treatment with resveratrol increased the number of taxa in the fecal microbiome of *db*/*db* mice, including *Bacteroides*, *Alistipes*, and *Alloprevotella*. Furthermore, it led to a decrease in LPS and inflammatory cytokines, such as TNF-α and IFN-γ, in the serum and intestines of mice. In an effort to corroborate the mechanism of the gut microbiota, Cai et al. (143) found that transplantation of the fecal microbiome community modified by resveratrol brought about a similar alteration in the gut microbiota to that observed in *db*/*db* mice following resveratrol intervention. In addition, it was also observed that the mRNA levels of TNF-α, IL-6, IFN-γ, and IL-1β were diminished in the kidneys. Taken together, the gut microbiota could provide a corresponding therapeutic effect by mediating the elimination of inflammation.

### 5.2 Regulating the stability of the intestinal barrier

Intestinal barrier disruption has been observed to accelerate the progression of kidney disease (144). Nonetheless, stability of the intestinal barrier can successfully reduce inflammation and kidney damage. Lan et al. (141) found that DKD mice exhibited damage to the colonic mucosal barrier and that FTZPs can repair the integrity of the colonic mucosal barrier in DKD mice, showing a dose-dependent increase in the expression of the intestinal epithelial tight junction proteins, e.g., cldn-4, zo-1, E-cadherin, and occludin. The colonic mucosal barrier was dramatically repaired upon a shift in the gut microbiota from FTZP-intervened animals to DKD mice, indicating the crucial role that gut microbiota modulation plays in the control of the intestinal barrier. In addition, analogous effects were observed in another study. Sanziguben Tang, which is composed of four herbs [i.e., *Rosa laevigate* Michx, *Gynostemma pentaphyllum* (Thunb.) Makino, *Phyllanthus emblica* L., and *Schisandra chinensis* (Turcz.) Baill.], is an empirical prescription for the clinical treatment of DKD. It can reduce the expression of inflammatory factors by inhibiting the activation of NF-κB, thereby protecting kidney tissue (145).

### 5.3 Reducing the levels of uremic toxins and endotoxins

Excess LPS produced by Gram-negative bacteria can lead to increased intestinal permeability and further damage to the intestinal barrier. Its excess can also translocate into the circulation (146), where it in turn activates the NLRP3 inflammatory vesicles to trigger persistent inflammation (147). SZP can decrease the quantity of Gram-negative bacteria, causing an approximately 1.93-fold reduction in the LPS levels in DKD mice, further reducing renal inflammation and improving kidney function (137). IS, a uremic toxin generated by the gut microbiota fermenting proteins, translocates into the nucleus after binding to the aryl hydrocarbon receptor (AhR), which contributes to kidney inflammation and fibrosis (148). The Tangshen formula (TSF) contains seven TCMs: astragalus [*Astragalus membranaceus* (Fisch.) Bge.], burning bush [*Euonymus alatus* (Thunb.) Sieb.], rehmannia (*Rehmannia glutinosa* Libosch), bitter orange (*Citrus aurantium* L.), cornus (*Cornus officinalis* Sieb. Et Zuce), rhubarb (*Rheum palmatum* L.), and notoginseng [*Panax notoginseng* (Burk.) F.H. Chen]. It has the effect of replenishing qi and nourishing yin, which promotes blood circulation and dredges collaterals, and is an empirical prescription for the clinical treatment of DKD. A prospective, multicenter, double-blind, randomized controlled trial showed that TSF could reduce the 24-h urinary protein level and increase the glomerular filtration rate in patients with DKD (149). Modern pharmacological research has shown that TSF can regulate the gut microbiota in DKD rats, particularly decreasing hazardous bacteria, and can also reverse the serum IS and LPS levels, thereby ameliorating kidney lesions (150).

### 5.4 Improving renal fibrosis

FTZPs could significantly reduce the area of fibronectin-positive regions and collagen deposition in the renal interstitium of DKD mice and downregulate the levels of the fibrosis-related pathway proteins such as Fbn2 and Itgb3. Studies involving FMT modified by FTZPs have demonstrated decreases in the glomerular mesangial matrix expansion and fibrosis in mice, indicating a strong connection between the function of the gut microbiota and the anti-fibrotic mechanism of FTZPs (141).

### 5.5 Regulating metabolic activities

The gut microbiota is involved in the body’s metabolic activities and in modulating illnesses by means of metabolites. For instance, SCFAs can suppress inflammatory responses and oxidative stress and enhance immune tolerance and are essential for the progression of CKD (151). FTZPs could increase the SCFA contents in the cecum of DKD mice and enhance the level of the SCFA transporter Slc22a19, thereby reducing kidney inflammation and damage (143). Moutan Cortex (MC) has the effect of clearing heat and cooling the blood, promoting blood circulation and removing blood stasis, while Moutan Cortex polysaccharide (MC-Pa) is one of the main active ingredients from MC. It can have antioxidant activity, can downregulate the expression of inflammatory factors, and can delay the progression of DKD (152). High doses of MC-Pa could increase the acetate, propionate, and butyrate levels by dynamically modulating the rat gut microbiota, which can help enhance the intestinal barrier function and decrease the pro-inflammatory mediators (153). Bile acids (BAs) transformed by the gut microbiota in the intestines can accumulate in the circulation, causing oxidative stress and inflammatory responses, which are major pathogenic factors of kidney damage and are associated with CKD (154). As a TCM prescription, QiDiTangShen (QDTS) granules have been used in the clinical treatment of DKD for several years. It is based on Zhang Jingyue’s theory of “Zhenyin Jingqi” and combined with clinical experience. It is composed of seven herbs: *R. glutinosa*, *Astragalus propinquus*, *Euryale ferox*, *C. officinalis*, *Whitmania pigra*, *Rheum officinale*, and *Hedyotis diffusa*. QDTS significantly reduced proteinuria in *db*/*db* mice, inhibited autophagy activity, and improved kidney pathological changes (155). A study found that QDTS could regulate *Lactobacillus*, *Bacteroides*, and *Roseburia* related to BA metabolism, reducing the serum BA levels in *db*/*db* mice to protect kidney function (156). Magnesium lithospermate B (MLB) could regulate the disordered gut microbiome and inhibit the increase in the total BA levels in mice, affecting BA deconjugation to prevent kidney damage (157). Furthermore, TCM believes that corn silk is a diuretic with diuretic and detumescent effects. It can be used to treat edema, proteinuria, urinary tract infection, and diabetes, and modern pharmacological research believes that it has antioxidant effects and is beneficial for the prevention and treatment of DKD (158). Corn silk polysaccharides (CSPs) have been observed to improve metabolic disorders to limit kidney damage in DKD rats by modifying the structure of the gut microbiota, specifically by enhancing the structure of endogenous metabolites (159).

The pathways linked to CSPs include glycerophospholipid, BA, tyrosine, tryptophan, fatty acid, and phenylalanine metabolism, which show strong correlations with the gut microbiota, such as *Firmicutes*, Bacteroidota, Lachnospiraceae_NK4A136_group, and *Dubosiella*. In recent years, the application of TCM in the solid-state fermentation of edible fungi has attracted more and more attention. *Astragalus* and its preparations have been shown to have a positive effect on the treatment of kidney disease (160). Microbial fermentation of TCM can improve its efficacy through microbial biotransformation. Natural *Cordyceps cicadae* is widely used in TCM to treat CKD, and *Paecilomyces cicadae*, as the anamorph stage of *C. cicadae*, can be used as its substitute. Modern studies have discovered that Radix astragali and *P. cicadae*-fermented (RPF) can improve the renal structure of DKD and reduce podocyte apoptosis, which is better than *A. membranaceus* (161). Qing Zhou’s research found that RPF could effectively lower the critical disease parameters, including urine protein, serum creatinine, and blood urea nitrogen, in DKD mice by reversing gut microbiota dysbiosis, in which the eight species of bacteria reversed by RPF correlated significantly with the biochemical indicators and related metabolic parameters in DKD (162). Huangkui capsule is a Chinese patent medicine made from the ethanol extract of the flowers of *Abelmoschus manihot*. Its pharmacologically bioactive ingredients include rutin, hyperoside, hibifolin, isoquercetin, myricetin, quercetin, and quercetin-3-*O*-robinobioside, which can be used for the treatment of DKD, CKD, and IgA nephropathy and have the effects of improving renal function and reducing proteinuria (163). Shi et al. (164) found that the alterations in the gut microbiota of DKD mice were related to the changes in the plasma metabolites and that intervention with the Huangkui capsule brought about the restoration of the intestinal microbiota and the amelioration of the metabolite levels in DKD mice, ultimately exerting the effect of delaying the further deterioration of DKD.

## 6 Discussion

NS can bring about abnormalities in the gut microbiota, which in turn impact the onset and progression of NS. Analysis of the pathogenesis of NS from the perspective of the gut microbiota can offer fresh avenues for clinical treatment and drug development. This holds immense significance for the prevention and cure of NS. Intervention of the gut microbiota can be complementary to NS therapeutic approaches and posits considerable value for clinical research. Current therapies that target the gut microbiome, such as probiotic therapy and FMT, represent encouraging prospects for NS treatment.

TCM has certain advantages in regulating the gut microbiota to treat NS, which is capable of modulating the internal environment to reduce proteinuria and protect kidney function. However, there are many issues and limitations in the process of improving the gut microbiota with TCM. To begin with, numerous clinical trials suffer from poor design, and there is a lack of large-scale and high-quality studies to clarify what specific flora are acting as therapeutic NS agents in TCM. Furthermore, due to the complexity and holistic nature of the gut microbiota, it is difficult to explain the potential mechanisms of treatment through a single flora. This also hinders the discovery of potential signaling pathways and therapeutic targets. All in all, current research on the treatment perspective of the gut microbiota is still at a very early stage of development, with various research aspects yet to mature.

The method of treating NS by improving the gut microbiota with TCM is challenging, but is fully promising. Future research into TCM that aims to regulate the gut microbiota to treat NS should continue to conduct multicenter, large-scale clinical trials in order to discover constructive solutions in practice.
